# Optimising data curation pipelines for population-level analytics in secure data environments: Findings from a phenome-wide analysis in the NHS England Secure Data Environment

**DOI:** 10.23889/ijpds.v8i2.2326

**Published:** 2023-09-14

**Authors:** Marwa Al Arab, Zach Welshman, Elias Allara, John Nolan, Rachel Denholm

**Affiliations:** 1 University of Bristol, Bristol, United Kingdom; 2 HDR UK, London, United Kingdom; 3 University of Cambridge, Cambridge, United Kingdom

## Objectives

Secure data environments (SDE) ensure safe access to large population-level sensitive data. However, computational capacity is limited in these environments, which leads to challenges in the analysis of large population data within the constraints of a complex cloud architecture leveraging multiple software ecosystems. Here we present an efficient pipeline to conduct phenome-wide analyses using electronic health records (EHR) in the NHS England SDE.

## Methods

We accessed deidentified linked EHR from NHS SDE for around 50 million people in England. The exposure is SARS-CoV-2 infection, with outcomes being a phenome-wide atlas of all diseases recorded in EHR data. For computational efficiency, we created three cohorts tables using PySpark within the Databricks environment and a sampling algorithm with inverse probability weights which adds a flag to the dataset to mark the inclusion of a row in the sample of a specific outcome. We will conduct survival analysis using Cox models in RStudio on the samples while adjusting for potential confounders in the main datasets and 15 subgroups.

## Results

Sampling with inverse probability weighting produced datasets that are statistically equivalent to the original population data. In terms of computational efficiency, the time needed to sample and read the data for modeling one outcome is 2.3 min compared to ~45 min when trying to read the entire dataset, which could fail due to the 4GB memory limits of in Rstudio within the SDE. This is particularly important in our study since we will be running at least 13,296 models for main and subgroup analysis in the three cohorts. By adding a flag to each data row to indicate its inclusion in a sample, the sampling strategy significantly reduced the storage space required for the outcome table of each sample.

## Conclusion

Preparing datasets in Databricks and applying sampling can increase the efficiency of big data analysis pipelines within SDE, save storage space, and help in avoiding memory overload caused by using complete datasets for statistical analysis.

